# Knowledge, Attitude, and Practice of Healthcare Providers Toward Novel Coronavirus 19 During the First Months of the Pandemic: A Systematic Review

**DOI:** 10.3389/fpubh.2021.606666

**Published:** 2021-06-25

**Authors:** Gobezie T. Tegegne, Belayneh Kefale, Melaku Tadege Engidaw, Amsalu Degu, Desalegn Tesfa, Amien Ewunetei, Taklo Simeneh Yazie, Mulugeta Molla

**Affiliations:** ^1^Department of Pharmacology and Clinical Pharmacy, School of Pharmacy, College of Health Sciences, Addis Ababa University, Addis Ababa, Ethiopia; ^2^Clinical Pharmacy Unit and Research Team, Department of Pharmacy, College of Health Sciences, Debre Tabor University, Debre Tabor, Ethiopia; ^3^Department of Social and Public Health, College of Health Sciences, Debre Tabor University, Debre Tabor, Ethiopia; ^4^Department of Pharmaceutics and Pharmacy Practice, School of Pharmacy and Health Sciences, United States International University-Africa, Nairobi, Kenya; ^5^Pharmacology Unit and Research Team, Department of Pharmacy, College of Health Sciences, Debre Tabor University, Debre Tabor, Ethiopia

**Keywords:** COVID-19, knowledge, attitude, practice, healthcare provider

## Abstract

**Introduction:** Coronavirus disease (COVID-19) is a highly contagious viral infection that has spread to every corner of the world. Lack of knowledge among healthcare providers (HCPs) about diseases such as COVID-19 may delay the diagnosis, disease spread, and produce poor infection control practices. Hence, this systematic review aimed to summarize the knowledge, attitudes, and practices (KAP) of HCPs toward COVID-19 during the first months of the pandemic.

**Methods:** A systematic review was conducted according the PRISMA guidelines, and the protocol was registered on PROSPERO (CRD42020191742). A relevant article search was performed on EMBASE, PubMed, CINAHL, Scopus, and the Google Scholar database. The methodological quality of studies was assessed using the Newcastle-Ottawa Quality Assessment Scale. The median percentage of HCPs with good KAP was computed.

**Results:** Twenty studies involving 12,072 HCPs were included in the review process. Median percentages of 75.8% (IQR: 69.3–87.7%), 74.6% (IQR: 54.4–74.6), and 79.8% (IQR: 67.0–79.8%) of HCPs had good knowledge, and positive attitude and practice, respectively. Although the reported risk factors were inconsistent among studies, age, gender, level of education, experience, infection prevention training, and sources of information were associated with knowledge of HCPs. In addition, being elderly, having a high level of education, absence of chronic illness, and good knowledge and practice were significantly associated with the attitude of HCPs. Further, types of profession, experience, age, level of education, use of personnel protective equipment, and gender were significantly associated with the practice of HCPs.

**Conclusions:** Approximately, three-fourths of HCPs had good knowledge, attitudes, and practices toward COVID-19 during the first months of the pandemic, although the percentage of HCPs was inconsistent in different study settings. In addition, associated factors of KAP were inconsistent among studies; hence, stake holders should target locally identified risk factors to design relevant education packages and infection prevention training to halt the rapid transmission of COVID-19.

**Systematic Review Registration:** https://www.crd.york.ac.uk/prospero/display_record.php?ID=CRD42020191742, identifier: CRD42020191742.

## Introduction

The world experienced previous epidemics and pandemics such as prehistoric epidemics, the black death, the Spanish Flu, HIV/AIDS, the H1N1 Swine Flu pandemic, the Ebola and the Zika Virus outbreaks. The 1918 influenza pandemic, caused by the H1N1 virus, was the most severe in recent history, and infected one-third of the world's population, and killed at least 50 million people worldwide (1).

The recent Coronavirus pandemic is a highly contagious, pathogenic viral infection that has spread globally at an unprecedented rate. The virus has spread to over 215 countries, affecting more than 110 million people, at the time of writing this review, and has so far been responsible for more than 2.4 million deaths worldwide (2). The WHO declared COVID-19 infection a global pandemic on March 11, 2020. Since then, healthcare authorities all around the world have launched awareness-raising and preparedness programs (3).

Amidst the current pandemic, the WHO has issued several guidelines, online courses, and training sessions to improve HCP's KAP about COVID-19 infection prevention and control. HCPs are frontline workers, who have close contact with the community; hence, a better knowledge of the disease is essential to halt the rapid and widespread transmission within health care settings and communities. A poor understanding of the disease among HCPs may result in delayed diagnosis, treatment, the rapid spread of the disease, and poor infection control practices. Hence, the principle of zero occupational infection-risk (4) and community transmission remain unachievable. Further, different factors contribute to better knowledge, attitudes, and practices among HCPs, however, there has been no published review in which the extent of KAP and associated factors among HCPs has been investigated. Thus, in this study, we aimed to investigate the level of KAP and associated factors among HCPs about COVID-19. The findings from this systematic review can be a guide to develop relevant training and courses about the current pandemic and the future.

## Methods

A systematic review was performed using the PRISMA flow diagram (5). The review protocol was registered in PROSPERO (6). The PRISMA checklist was also strictly adhered to while conducting this systematic review (7).

### Data Sources and Search Strategy

A systematic search was conducted by reviewing legitimate databases and indexing services. Accordingly, PubMed, Cumulative Index to Nursing and Allied Health Literature (CINAHL), Scopus, EMBASE (Ovid), and other supplementary sources, including Google Scholar, Cochrane library, and Research Gate were used. Advanced search strategies were applied in major databases to retrieve relevant findings closely related to the knowledge, attitudes towards, and practices of HCPs in relation to COVID-19. The search was conducted with the aid of carefully selected key-words and indexing terms. These include keywords related to “COVID-19,” “Knowledge,” “Attitude,” “Practice,” and “Healthcare providers.” Boolean operators (“OR,” “AND”) and truncation were used to identify relevant articles and records that met our research question. The search was conducted from 20th April−10th May 2020, and all published articles about the KAP of HCPs on COVID-19 were included in the systematic review. Bibliography lists from all eligible articles were also hand-searched to identify additional papers that could potentially be relevant for inclusion.

### Inclusion and Exclusion Criteria

All observational studies published in English, in which knowledge, attitudes, and practices of HCPs toward COVID-19 were included in the review. Articles related to Middle East respiratory syndrome coronavirus (MERS-COV) and severe acute respiratory syndrome-related coronavirus (SARS-CoV) were excluded. Besides, articles that contained insufficient information; records with missing outcomes of interest; and findings from personal opinions, editorial reports, letters to the editors, case reports and series, unpublished articles, systematic reviews, and qualitative studies were also excluded from the review.

### Article Screening Process

Records identified from various electronic databases, indexing services, and directories were saved and exported to Covidence software. Duplicate records were removed using Covidence software. Some duplicates were addressed manually due to variations in reference styles across sources. The initial title and abstract screening were done by the two authors (BK and GT). Three categories (yes, no, maybe) were used during the selection process. The full text of studies considered “yes” or “maybe” during the screening was assessed based on the eligibility criteria by two authors (GT and BK). Then, full-text screening was conducted by two authors (ME and DT). In each case, the third author (AD) played a critical role in solving discrepancies that arose between two authors.

### Data Extraction and Synthesis

Two authors (MM and TY) independently extracted relevant data using a standardized data abstraction format prepared in Microsoft Excel. The extracted data included study characteristics (country and study setting, first author, publication year, study design, population characteristics, and sample size) and the result of studies (the percentage of HCPs with good knowledge, attitude, practice, and contributing factors). Any disagreements were resolved through discussions with the third author (AE) and by cross-checking the papers.

### Methodological Quality Assessment of Studies

The methodological quality and risk of bias of the included studies were independently assessed by two authors (BK and MT) using the Newcastle-Ottawa scale (8), which rates study quality out of 10 points (stars). For ease of evaluation, the tool included important indicators categorized in to three major domains. The first section assesses the methodological quality of a study, which has a maximum of five stars. The second section considers the comparability of the study and takes two stars, and the remaining section is used to assess the outcomes of studies with related statistical analysis. This critical assessment was conducted to assess the internal and external validity of the included studies. As indicated in Table 1, the mean score of two authors (BK and ME) was taken for the final decision, and studies with a score greater than or equal to five points/stars were included (9, 10).

**Table 1:** Quality assessment of included studies using Newcastle-Ottawa scale.

**References**	**Methodological quality (4)**	**Comparability^**1**^**	**Outcomes measures and analysis (2)**	**Total (8)**
Shi et al. (13)	2	2	2	6
Gambhir et al. (25)	4	0	1.5	5.5
Saqlain et al. (15)	5	2	1.5	8.5
Taghrir et al. (14)	3	0	2	5
Kamate et al. (26)	3.5	2	2	7.5
Tadesse et al. (19)	3	0	2	5
Zhong et al. (16)	3	2	2	7
Olum et al. (17)	3	2	2	7
Gao et al. (31)	3	1	2	6
Alwani et al. (27)	4	0	2	6
Taneja and Khurana (12)	4	0	1	5
Nepal et al. (21)	3	1	2	6
Bhagavathula et al. (28)	3.5	0.5	2	6
Alhaj et al. (11)	2.5	0.5	2	5
Hamza et al. (29)	4.5	2	1.5	8.5
Karasneh et al. (30)	3	0	2	5
CAMARA (20)	3	1.5	2	6.5
Alrubaiee et al. (22)	3.5	0.5	1.5	5.5
Muhammad et al. (24)	4.5	2	2	8.5
Ayinde et al. (23)	4	2	2	8

### Outcome Measurements and Data Analysis

The outcome measurements of this systematic review were reported in terms of the percentage of HCPs with good knowledge, attitudes, and practices toward COVID-19. As studies used variable KAP assessment tools, median and quartiles were computed. Further, contributing factors were also summarized in a table as socio-demographic and other characteristics.

## Results

### Study Selection

**Figure 1:**
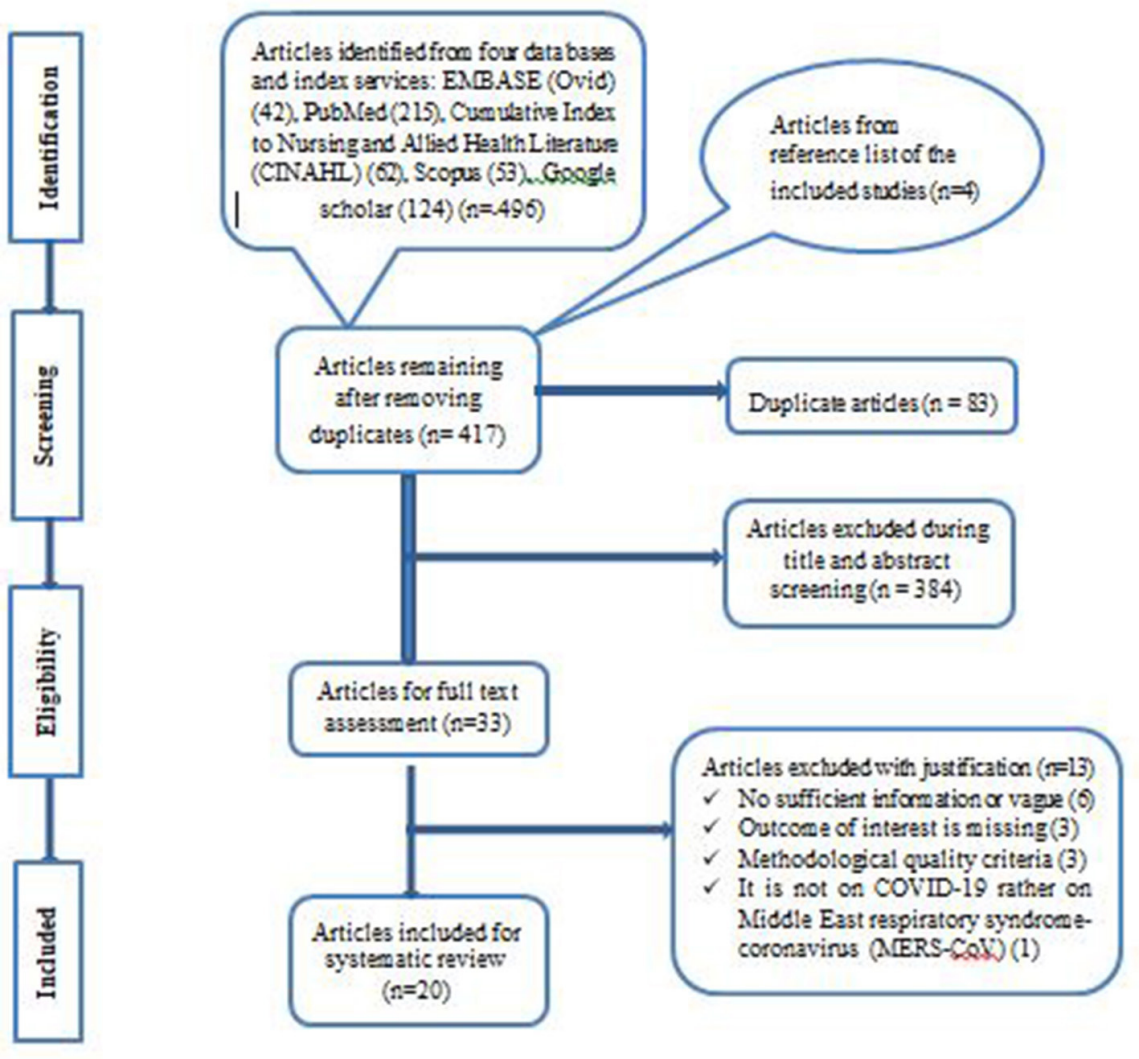
PRISMA flow chart of the study selection process.

The literature search identified a total of 502 records from several sources. After the removal of duplicate records using Covidence software, the remaining 419 records were screened, and 386 records were excluded. The remaining 33 full-text records were then evaluated as per the predetermined eligibility criteria. Thirteen articles were also excluded due to insufficient or vague information (6), missing outcome of interest (3), poor methodological quality (3), and not related to the novel COVID-19 (1). Finally, 20 articles met the eligibility and study quality assessment criteria and were included in this systematic review (Figure 1).

### Characteristics of the Included Studies

A total of 20 studies with 12,072 health professionals were included in this systematic review. All the included studies employed a cross-sectional study design. The number of study participants ranged from 52 (11) in a multi-nation study to 3,595 (12) HCPs in India. All studies (*n* = 20) reported knowledge, while 12 (13–24) and 12 (14–17, 19, 21–26) studies included reported the percentage of HCPs with positive attitudes and practices toward COVID-19, respectively. In addition, nine studies (14–17, 21–24) were included in which the knowledge, attitudes, and practices of HCPs were investigated, while six (11, 12, 27–30), three (13, 18, 20), and two (25, 26) studies included only knowledge, knowledge and attitude only, knowledge and practice only, respectively. The percentage of HCPs with good knowledge, positive attitude, and practice ranged from 30.2% in India (25) to 93.2% in Pakistan (15), 21% in Uganda (17) to 93.2% in in Vietnam (31), and 57.3% in Pakistan (24) to 94.47% in Iran (14), respectively. Regarding study setting, 12 studies were conducted in Asia (12–15, 18, 21, 22, 24, 25, 27, 30, 32), five in Africa (17, 19, 20, 23, 29), and three were conducted in more than one continent (11, 26, 28) (Table 2). The methodological quality scores of all studies ranged from 5 to 8.5 as per the Newcastle-Ottawa scale (Table 1).

**Table 2:** Characteristics of the included studies in the systematic review.

**References**	**Study country**	**Study design**	**Study participants**	**Sample size**	**Knowledge *N* (%)**	**Attitude *N* (%)**	**Practice *N* (%)**
Shi et al. (13)	China	CS	Psychiatrists and psychiatric nurses	311	278 (89.51)	240 (77.17)	NR
Gambhir et al. (25)	India	CS	Dentist	215	65 (30.2)	NR	135 (62.8)
Saqlain et al. (15)	Pakistan	CS	Doctors, pharmacists, nurses	414	386 (93.2)	344 (83.1)	367 (88.7)
Taghrir et al. (14)	Iran	CS	Medical students	240	208 (86.96)	122 (51)	227 (94.47)
Kamate et al. (26)	Multi-national	CS	Dentist	860	797 (92.7)	NR	683 (79.5)
Tadesse et al. (19)	Ethiopia	CS	Nurse	415	307 (74)	299 (72)	278 (67)
Zhou et al. (32)	China	CS	Doctors, nurses, paramedics	1,357	1,207 (89)	1,153 (85)	1,217 (89.7)
Olum et al. (17)	Uganda	CS	Nurse, midwife, doctors, medical officer, senior house officer	136	94 (69)	29	101 (74)
Huynh et al. (18)	Vietnam	CS	Physician, nurse, pharmacist and technical staff	327	289 (88.4)	305 (93.3)	NR
Alwani et al. (27)	Pakistan	CS	Nurse	85	66 (77.6)	NR	NR
Taneja and Khurana (12)	India	CS	Homeopathic practitioners	3,595	2,876 (80)	NR	NR
Nepal et al. (21)	Nepal	CS	Doctors, nurses, health assistant, others	353	290 (82.15)	321 (90.93)	295 (83.57)
Bhagavathula et al. (28)	Multi-national	CS	Doctors, medical students, pharmacists, academic doctor, nurse, lab technicians, dentists	453	284 (62.7)	NR	NR
Alhaj et al. (11)	Multi-national	CS	Neurosurgery residents	52	31 (60)	NR	NR
Hamza et al. (29)	Egypt	CS	Pharmacy students	238	175 (73.5)	NR	NR
Karasneh et al. (30)	Jordan	CS	Pharmacists	486	198 (40.4)	NR	NR
CAMARA (20)	Guinea	CS	Doctors, nurses, pharmacists, specialist physicians, students, biologists	548	387 (70.6)	317 (57.7)	NR
Alrubaiee et al. (22)	Yemen	CS	Physicians, nurses, laboratory, anesthesia, dentist, medical academician, pharmacist, midwifery	1,244	866 (69.6)	1,057 (85)	1,090 (87.6)
Muhammad et al. (24)	Pakistan	CS	Pharmacists	393	281 (71.5)	175 (44)	225 (57.3)
Ayinde et al. (23)	Nigeria	CS	Nurses, doctors, health attendant/ward maid, pharmacist	350	275 (78.6)	224 (64)	279 (79.8)

*CS, cross-sectional; NR, not reported*.

### Study Outcome Measures

#### Percentage of HCPs With Good Knowledge, Attitude, and Practice

**Figure 2:**
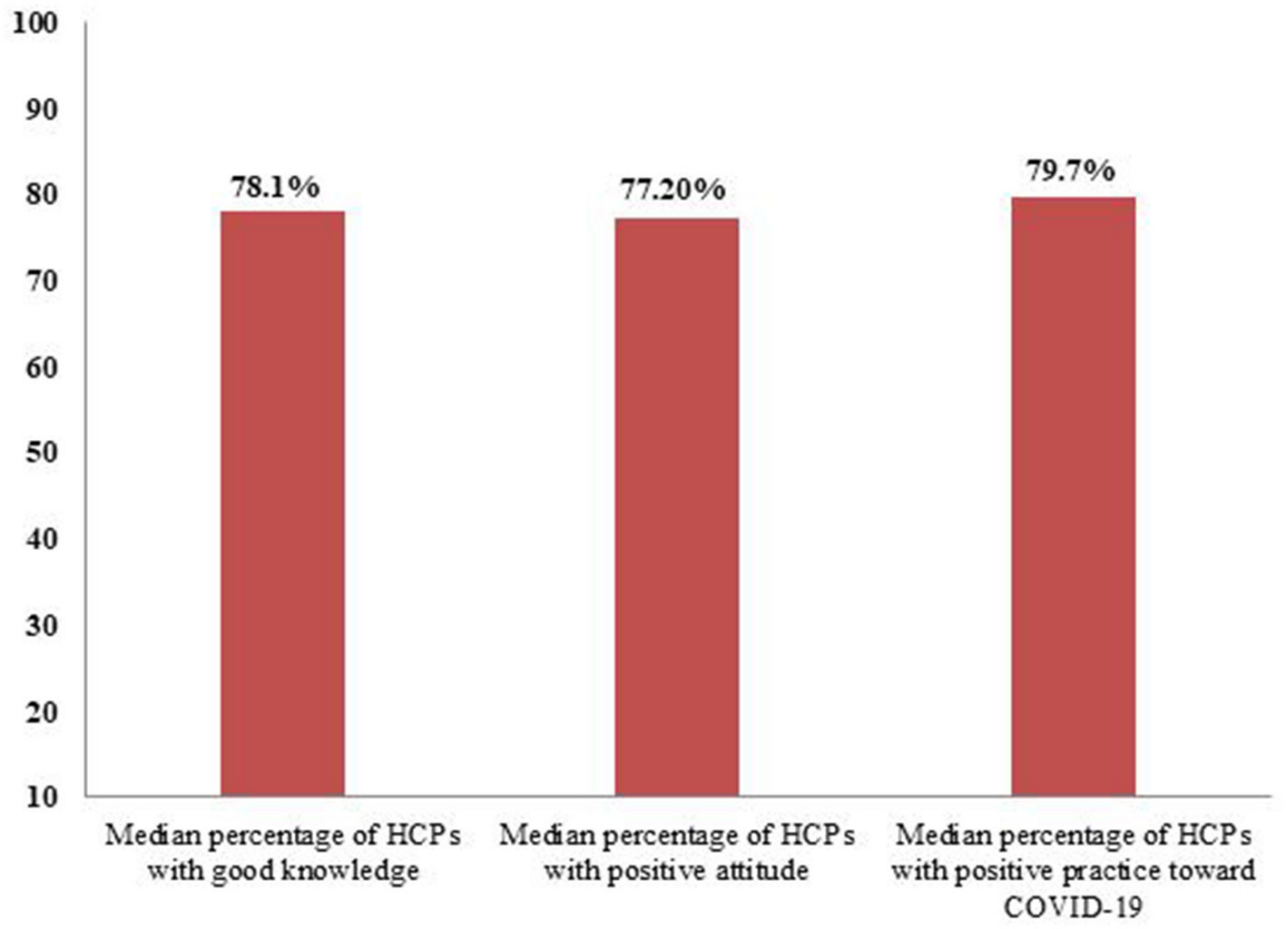
Median percentage of HCPs with good knowledge and positive attitude and practice toward COVID-19.

The median percentage of HCPs with good knowledge among 20 studies was 75.8% (IQR: 69.3–87.3%). In addition, the percentage of HCPs with good knowledge was variable and ranged from 30.2–93.2%. Considering the attitude of HCPs, a median of 74.6% (IQR: 54.4–74.6) HCPs had a positive attitude about COVID-19 prevention control. A lesser percentage of 21.0% HCPs and a larger percentage of 93.3% HCPs were reported to have a positive attitude toward COVID prevention. Furthermore, a median of 79.8% (67.0–79.8%) HCPs had positive practice toward COVID-19. The highest percentage of 94.5% of HCPs practiced positively toward COVID (Figure 2).

#### Factors Associated With Knowledge, Attitudes, and Practices of Healthcare Providers Toward COVID-19

In this systematic review, various associated factors of knowledge, attitude, and practice of HCPs toward COVID-19 were identified. Factors associated with knowledge include age (15, 17), level of education (25), gender (20, 23, 24), service years (23, 26), types of profession (20, 21), sources of information (17), and taking infection prevention training (20, 21). The direction of the association of gender, service year, and taking infection prevention training were reported inconsistent among studies. Three studies reported that being female had a positive association with knowledge (20, 23), while one study reported the opposite (24). In addition, being elder, having a higher level of education, and the use of social media as sources of information were factors found to be associated with better knowledge. Regarding factors associated with attitude, having high knowledge (21, 24), absences of chronic illness, and a high level of education (24) were positively associated with attitude. Furthermore, types of profession (15, 21), service years (15), age (17), level of education (17), use of personal protective equipment (21), and gender (29) were significantly associated with HCPs' practice toward COVID-19 (Table 3).

**Table 3:** Factors associated with healthcare providers' knowledge, attitude, and practice toward COVID-19.

**References**	**Determinants of knowledge**	**Signs of association**	**Determinants of attitude**	**Signs of association**	**Determinants of practice**	**Signs of association**
Gambhir et al. (25)	Level of educational Engaged in academic and private practice	+ +				
Saqlain et al. (15)	Age	+			Service year Being pharmacist	+ +
Kamate et al. (26)	Service year Qualification	– +			Qualification	+
Olum et al. (17)	Age Information from News media (TV, radio, newspaper)	+ +			Age Level of educational	+ –
Nepal et al. (21)	Being doctors and nurses Taking infection prevention training	+ +	Knowledge score Practice score	+ +	Being doctor, nurse and health assistant Use of PPE Attitude score Knowledge score	+ + + +
Alhaj et al. (11)	Program location in North America, Europe and Arabian Gulf Cooperation Council countries.	+				
Hamza et al. (29)					Being female Lives in capital	+ –
Camara (20)	Being female IPC training in the past Being doctor, pharmacist and specialist physician	+ – –				
Muhammad et al. (24)	Being female	–	Age Level of education Level of concern Knowledge	++++		
Ayinde et al. (23)	Being female Service year Working in a secondary or tertiary institutions	+ + +				

## Discussion

The present systematic review aimed to synthesize the knowledge on the KAP of HCPs and their associated factors about COVID-19. After a thorough and meticulous search of the literature, 20 articles that met the inclusion criteria were identified. All of the studies were published in different medical journals and readily available for medical practitioners and decision-makers. In addition, our findings showed that there was a wider variability among studies. Further, around one-fifth of the included studies had a high methodological quality (>8 out of 10), indicating that most of the studies had a low risk of bias. This systematic review found that the median percentage of HCPs with good knowledge and positive attitude and practice about COVID was 75.8% (IQR: 69.3–87.7%), 74.6% (IQR: 54.4–74.6), and 79.8% (IQR: 67.0–79.8%), respectively.

Compared to previous studies conducted on epidemics such as the Ebola virus (33, 34), there was a higher knowledge gap among HCPs. This may be due to the rapid and robust activities of WHO on the current pandemics. Despite this, the number of individuals who got COVID is increasing daily. Therefore, comprehensive health awareness initiatives and training opportunities should be expanded and made easily accessible for HCPs to halt the rapid and widespread transmission of COVID-19.

This comprehensive literature review also identified factors associated with the KAP of HCPs toward COVID-19. These findings provide a scientific basis for developing a comprehensive multidimensional program that incorporates these factors to increase the KAP of health professionals, especially frontline workers. Some of the reported associated factors were inconsistent among studies, such as; gender, service year, and types of professions, and taking infection prevention training. Two studies (20, 23) showed that females had better knowledge than males, while another study found the opposite result. This disparity can be explained by the heterogeneity of the study participants in terms of the type of profession and years of experience. In summary, no clear conclusion can be drawn on the impact of gender, year of service, types of profession, and status of infection prevention control training. Hence, further studies are required to determine the correlation between these variables and knowledge.

On the other hand, the finding of the present reviewed studies consistently showed that health professionals who were older, highly educated, and who had obtained information from social media, such as television, had good knowledge of COVID-19 compared to their counterparts (24). A possible explanation for this finding is the ability of highly educated health professionals to easily understand the nature of the disease and identify the sources of information.

There is a need to develop a system of continuous education and reinforcement of the principles of standard precautions and hygiene to retain knowledge regarding COVID-19. To reduce the incidence and spread of infections, especially on the frontline workers, compliance with interventions is mandatory (35, 36). Therefore, WHO and other concerning bodies should pay more attention and support to the less informed HCPs with COVID-19 who need extensive training and information about the current pandemic.

In addition, HCPs with good knowledge, a higher level of education, and had no history of chronic disease had a better positive attitude toward COVID-19. Having good knowledge impacts the improvement of behavior and makes HCPs strictly follow the infection prevention guidelines (37). Despite the reported factors were infrequent, being pharmacists, doctors and nurses, year of service, elder, female, knowledgeable, user of personal protective equipment had significantly associated with a high level of better practice toward COVID-19.

Despite the fact that our review has several strengths, such as meticulous and complete search of published studies, some limitations need to be considered when interpreting the results. Firstly, there was a higher heterogeneity among the included studies; hence, readers should interpret the finding cautiously. Secondly, included studies used different KAP assessment tools, and hence, it was very challenging to summarize the KAP of HCPs. Thirdly, the HCPs targeted in each study were different, so this creates problems in determining the median percentage.

## Conclusions

Approximately, three-fourths of the healthcare providers had good knowledge and positive attitude and practice toward COVID-19 during the first months of the pandemic. Age and level of education were significantly associated with knowledge, attitude, and practice, while healthcare provider experience was found to be significantly associated with knowledge and practice only. This study has many implications for HCPs, policymakers, and other stakeholders. It provides a framework to establish interventions to improve the KAP of HCPs, especially those who work at the frontline. Emphasis should be given to empower frontline HCPs through education and training, as this is a key measure to avoid diagnosis delay, disease spread, and poor practice of infection control.

## Data Availability Statement

The original contributions presented in the study are included in the article/supplementary material, further inquiries can be directed to the corresponding author/s.

## Author Contributions

BK, GT, and ME contributed to conceptualization, data curation, investigation, formal analysis, methodology, writing of the original draft, review, and editing of the manuscript. DT, AD, TY, AE, and MM were involved in conceptualization, writing of the original draft, investigation, and methodology. All authors approved the submitted version of the manuscript critically.

## Conflict of Interest

The authors declare that the research was conducted in the absence of any commercial or financial relationships that could be construed as a potential conflict of interest.

## Abbreviations

CI: confidence interval
CINAHL: Cumulative Index to Nursing and Allied Health Literature
COVID-19: Coronavirus disease 19
HCP: Health Care provider
KAP: knowledge Attitude Practice
MERS-COV: Middle East respiratory syndrome coronavirus
PRISMA: Preferred Reporting Items for Systematic Reviews and Meta-Analyses
WHO: World Health Organization.
